# Thermodynamic and Kinetic Modeling of Co-utilization of Glucose and Xylose for 2,3-BDO Production by *Zymomonas mobilis*

**DOI:** 10.3389/fbioe.2021.707749

**Published:** 2021-07-26

**Authors:** Chao Wu, Ryan Spiller, Nancy Dowe, Yannick J. Bomble, Peter C. St. John

**Affiliations:** ^1^Biosciences Center, National Renewable Energy Laboratory, Golden, CO, United States; ^2^National Bioenergy Center, National Renewable Energy Laboratory, Golden, CO, United States

**Keywords:** dynamic flux balance analysis, thermodynamics analysis, enzyme protein cost analysis, kinetic models, *Zymomonas mobilis*, biofuel production, 23-butanediol

## Abstract

Prior engineering of the ethanologen *Zymomonas mobilis* has enabled it to metabolize xylose and to produce 2,3-butanediol (2,3-BDO) as a dominant fermentation product. When co-fermenting with xylose, glucose is preferentially utilized, even though xylose metabolism generates ATP more efficiently during 2,3-BDO production on a BDO-mol basis. To gain a deeper understanding of *Z. mobilis* metabolism, we first estimated the kinetic parameters of the glucose facilitator protein of *Z. mobilis* by fitting a kinetic uptake model, which shows that the maximum transport capacity of glucose is seven times higher than that of xylose, and glucose is six times more affinitive to the transporter than xylose. With these estimated kinetic parameters, we further compared the thermodynamic driving force and enzyme protein cost of glucose and xylose metabolism. It is found that, although 20% more ATP can be yielded stoichiometrically during xylose utilization, glucose metabolism is thermodynamically more favorable with 6% greater cumulative Gibbs free energy change, more economical with 37% less enzyme cost required at the initial stage and sustains the advantage of the thermodynamic driving force and protein cost through the fermentation process until glucose is exhausted. Glucose-6-phosphate dehydrogenase (g6pdh), glyceraldehyde-3-phosphate dehydrogenase (gapdh) and phosphoglycerate mutase (pgm) are identified as thermodynamic bottlenecks in glucose utilization pathway, as well as two more enzymes of xylose isomerase and ribulose-5-phosphate epimerase in xylose metabolism. Acetolactate synthase is found as potential engineering target for optimized protein cost supporting unit metabolic flux. Pathway analysis was then extended to the core stoichiometric matrix of *Z. mobilis* metabolism. Growth was simulated by dynamic flux balance analysis and the model was validated showing good agreement with experimental data. Dynamic FBA simulations suggest that a high agitation is preferable to increase 2,3-BDO productivity while a moderate agitation will benefit the 2,3-BDO titer. Taken together, this work provides thermodynamic and kinetic insights of *Z. mobilis* metabolism on dual substrates, and guidance of bioengineering efforts to increase hydrocarbon fuel production.

## Introduction

*Zymomonas mobilis* is a facultative anaerobic Gram-negative microorganism, well known for its efficient production of bioethanol as replacement for fossil fuels (Swings and De Ley, 1977; Skotnicki et al., 1982; Sprenger, 1996; Gunasekaran and Raj, 1999; He et al., 2014; Yang et al., 2016a). The bacterium possesses a relatively simple central metabolic network, including a non-functional Embden-Meyerhof-Parnas (EMP) glycolytic route (Fuhrer et al., 2005; Seo et al., 2005; Felczak et al., 2019), incomplete pentose phosphate pathway (Feldmann et al., 1992; De Graaf et al., 1999) and truncated tricarboxylic acid (TCA) cycle (Lee et al., 2010; Jacobson et al., 2019). Instead, hexoses including glucose and fructose are metabolized *via* the Entner-Doudoroff (ED) pathway to form primarily ethanol, along with glycerol and succinic, lactic and acetic acid by-products (Zhang et al., 2019b). Because of its unusual and efficient metabolic machinery, *Z. mobilis* can achieve remarkably high bioprocess efficiency, with up to 98% of hexose carbon converted into ethanol (Kalnenieks et al., 2019). In recent decades, genetic engineering has been used to broaden the spectrum of fermentation substrates and products by *Z. mobilis*. Introduction of exogenous enzymes of xylose isomerase, xylulokinase, transketolase, and transaldolase from *Escherichia coli* endows the bacterium the capability of fermenting pentose sugars (Zhang et al., 1995), whereas *Enterobacter cloacae* derived acetolactate synthase, acetolactate decarboxylase and butanediol dehydrogenase help the redirection of carbon flux to 2,3-butanediol (2,3-BDO), a bio-derived precursor for gasoline and jet fuel (Syu, 2001; Celinska and Grajek, 2009; Ji et al., 2011; Yang et al., 2016b).

Metabolism begins with the uptake of sugar substrates from the media by the glucose facilitator protein, glf, in the cell membrane of *Z. mobilis*. Glf is a low-affinity, high-velocity carrier that also transports xylose but with the competitive inhibition by glucose (Dimarco and Romano, 1985). In fermentation, glucose is preferentially utilized by *Z. mobilis* when co-fermenting with xylose (Zhang et al., 1995). Stoichiometrically, 1 mol of glucose is consumed to form 1 mol of 2,3-BDO with 1 mol of ATP generated *via* the ED pathway as:

1glucose→2,3-BDO+2CO+2NADPH+ATP

For xylose, 1.2 mol of ATP will be produced when xylose is metabolized through the completed pentose phosphate pathway and lower glycolysis to produce an equal amount of 2,3-BDO:

1.2xylose→2,3-BDO+2CO+20.2NADH+0.8NADPH+1.2⁢ATP

**FIGURE 1:**
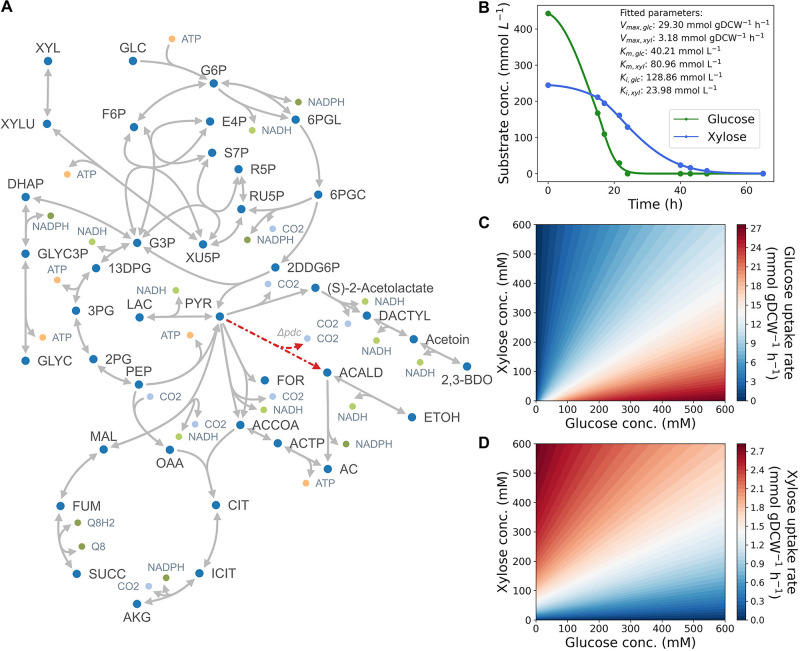
**(A)** Map of core-carbon metabolism in the engineered *Z. mobilis* strain. A list of abbreviations used are shown in Supplementary Table 1. **(B)** Kinetic properties of glucose facilitator (glf) in *Z. mobilis* BC42C Δpdc. Kinetic parameters (V_*max*_, K_*m*_, and K_*i*_) are estimated by fitting the time-course utilization of glucose (green) and xylose (blue) with coefficient of determination *R*^2^ = 0.99. Dots denote experimental data points, and lines denote the best fit curve of substrates uptake kinetics. **(C,D)** Contour plots illustrating substrate uptake rates as a function of glucose **(C)** and xylose **(D)** concentrations.

Yields of ATP from xylose are higher than for glucose on 2,3-BDO basis in large part because carbon from xylose is rearranged in the pentose phosphate pathway into glyceraldehyde-3-phosphate, whereas for glucose, only half of the carbon flows through glyceraldehyde-3-phosphate due to 2-dehydro-3-deoxy-phosphogluconate aldolase in the ED pathway (Figure 1A). Engineered xylose metabolism in *Z. mobilis*, therefore, more closely resembles xylose metabolism in organisms that utilize the EMP pathway. Accordingly, a bioenergetic question arises as to why an energy inefficient glucose metabolism pathway is preferred over xylose metabolism.

Max-min driving force (MDF) optimization and enzyme protein cost estimation have been proposed to evaluate to what extent a metabolic pathway’s flux is constrained by thermodynamic driving force and determine the protein expense required to support a pathway’s enzymes (Flamholz et al., 2013; Noor et al., 2014). Thermodynamically, the directionality and feasibility of a reaction is determined by the Gibbs free energy change, ΔG′, in which a large negative value indicates a strong driving force for the reaction to proceed forward. Similarly, the driving force of a metabolic pathway can be assessed by solving a maximin problem, which seeks to maximize the negative ΔG′ of the most thermodynamically unfavorable reaction by tuning the concentrations of involved metabolites (Flamholz et al., 2013; Noor et al., 2014; Wu et al., 2020). On the other side, the enzyme level required by a reaction to proceed is determined by both reaction thermodynamics and the kinetic properties of the enzyme, according to a common modular rate law (Liebermeister et al., 2010) and the Haldane relationship (Alberty, 2006). Accordingly, the total enzyme mass required to support a unit pathway flux can be estimated by solving a non-linear optimization with respect to intermediate concentrations (Flamholz et al., 2013; Wu et al., 2020). So far, the methodology has been successfully applied in the thermodynamic comparison between glycolytic pathways in *E. coli* (Flamholz et al., 2013; Noor et al., 2014), and photosynthesis pathways in cyanobacteria with thermodynamic bottlenecks and potential engineering targets identified to enhance CO_2_ fixation (Janasch et al., 2019; Wu et al., 2020).

In this work, we first use MDF and enzyme cost to investigate the thermodynamic and kinetic properties of co-utilization of glucose and xylose by a 2,3-BDO producing *Z. mobilis* strain BC42C Δpdc which diverts carbon flux from ethanol biosynthesis through a pyruvate decarboxylase knockout (Zhang and Himmel, 2019; Zhang et al., 2019a). Next, we extend the mathematical modeling from pathway level to the core metabolic network of *Z. mobilis* using dynamic flux balance analysis (dynamic FBA). With a validated model, optimization is performed to identify the optimal initial sugar ratio and agitation conditions for 2,3-BDO fermentation. This work reveals a deeper understanding of mixed C5/C6 sugar metabolism by *Z. mobilis* and sheds light on rational design of bioengineering and fermentation for hydrocarbon fuel production.

## Results

### Uptake Kinetics of Glucose and Xylose in *Zymomonas mobilis*

Following previous studies (Dimarco and Romano, 1985; Schoberth and de Graaf, 1993; Ren et al., 2009), we began by constructing a Michaelis–Menten kinetics model with competitive inhibition term for the dual-functional transporter in *Z. mobilis* (Materials and Methods). Kinetics parameters (V_*max*_, K_*m*_, and k_*i*_) for both glucose and xylose were estimated by fitting experimental results. As shown in Figure 1B, simulated kinetic curves match well with the data (*R*^2^ = 0.99). The fitting results suggest that the maximum transport capacity of glucose is ninefold higher than xylose (29.30 mmol gDCW^–1^ h^–1^ vs. 3.18 gDCW^–1^ h^–1^ in V_*max*_), and glucose is twofold more affinitive to the transporter than xylose (40.21 mM vs. 80.96 mM in K_*m*_), which could explain the preferential utilization of glucose when co-fermenting with xylose from a kinetics perspective. It is also noteworthy that the inhibitor constant of glucose was estimated to be much greater than that of xylose, which suggests that competitive inhibition might not be the dominant factor in substrate preference of *Z. mobilis*.

With the estimated kinetic parameters, we simulated glucose and xylose uptake rates at different glucose and xylose concentrations (Figures 1C,D). The contour plots illustrate how glucose and xylose uptake rates change as a function of concentrations of both substrates. In general, glucose uptake rate correlates positively with glucose concentration, and negatively with xylose concentration. Xylose uptake rate mirrors the pattern but lowered by almost an order of magnitude, determined by the maximum uptake capacity of the two substrates. Despite the low uptake rate of xylose, transport of this substrate still occurs in the initial phase of fermentation, which leads to simultaneous and not sequential utilization of glucose and xylose.

### Thermodynamic Driving Force and Enzyme Protein Cost of Glucose and Xylose Metabolism in *Zymomonas mobilis*

**FIGURE 2:**
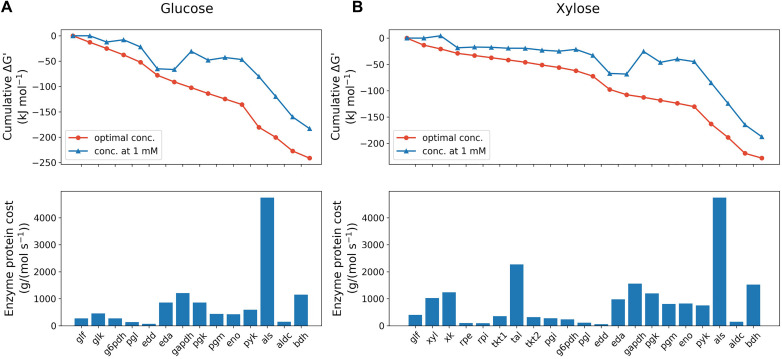
Max-min driving force (MDF) optimization (upper panels) and enzyme protein cost estimation (lower panels) of glucose **(A)** and xylose **(B)** metabolism pathway of *Z. mobilis* BC42C Δpdc at the initial state. MDF optimization is presented as the cumulative sum of reaction Gibbs free energies, ΔG′. Blue line with triangle symbols denotes standard Gibbs free energies with all metabolite concentrations fixed at 1 mM, and red line with circle symbols denotes Gibbs free energies when the minimal ΔG′ is optimized with metabolite concentrations constrained ranging from 1 μM to 10 mM except that extracellular glucose/xylose concentration fixed at measured value. Enzyme protein costs were optimized for the minimal protein mass required to support unit pathway flux under the same concentration constraints with MDF optimization. eda, 2-dehydro-3-deoxy-phosphogluconate aldolase; edd, phosphogluconate dehydratase; eno, enolase; g6pdh, glucose-6-phosphate dehydrogenase; gapdh, glyceraldehyde-3-phosphate dehydrogenase; glf, glucose facilitator protein; glk, glucokinase; pgi, glucose-6-phosphate isomerase; pgl, 6-phosphogluconolactonase; pgm, phosphoglycerate mutase; pgk, phosphoglycerate kinase; pyk, pyruvate kinase; rpe, ribulose-5-phosphate epimerase; rpi, ribose-5-phosphate isomerase; tal, transaldolase; tkt, transketolase; xk, xylulokinase; xyl, xylose isomerase.

Thermodynamic analysis was performed to compare the feasibility of glucose and xylose metabolism pathways. Kinetic parameters for substrate uptake were estimated from experimental data, while parameters for other pathway enzymes were taken from the Brenda database (Jeske et al., 2019). Three enzyme reactions catalyzed by glucose-6-phosphate dehydrogenase (g6pdh), glyceraldehyde-3-phosphate dehydrogenase (gapdh) and phosphoglycerate mutase (pgm) in the glucose utilization pathway were identified as thermodynamic bottlenecks for positive Gibbs free energy changes assuming 1 mM concentration of substrate and product (Figure 2). In addition to the three shared reactions, two more bottlenecks, xylose isomerase (xyl) and ribulose-5-phosphate epimerase (rpe), were found in the xylose pathway. The MDF of the initial phase of sugar fermentation was optimized for both pathways by tuning concentrations of involved metabolites with extracellular substrates fixed at measured concentrations. The results show that glucose metabolism is initially slightly more favorable (−241.2 vs. −228.0 kJ mol^–1^) than the xylose pathway thermodynamically.

Enzyme protein costs were estimated in both pathways, which calculate the total protein cost needed to support a unit pathway flux. As shown in Figure 2, the total protein requirement of xylose utilization pathway is 58% higher [1.9 × 10^4^ g/(mol s^–1^) vs. 1.2 × 10^4^ g/(mol s^–1^)] than glucose metabolism at the beginning of fermentation, which indicates a protein burden for *Z. mobilis* to initiate xylose metabolism when co-fermenting with glucose. It was also found that, likely due to the high K_*m*_ value, the exogenous enzyme acetolactate synthase (als) accounts for the dominant amount of protein cost in both pathways: 41% and 25% of total pathway protein requirement for glucose and xylose, respectively. Consistent with a previous study (Yang et al., 2016b), our results suggests that als is the bottleneck in acetolactate generation from pyruvate, and because the limitation is kinetic rather than thermodynamic, represents a potential metabolic engineering target to improve 2,3-BDO production in *Z. mobilis*.

**FIGURE 3:**
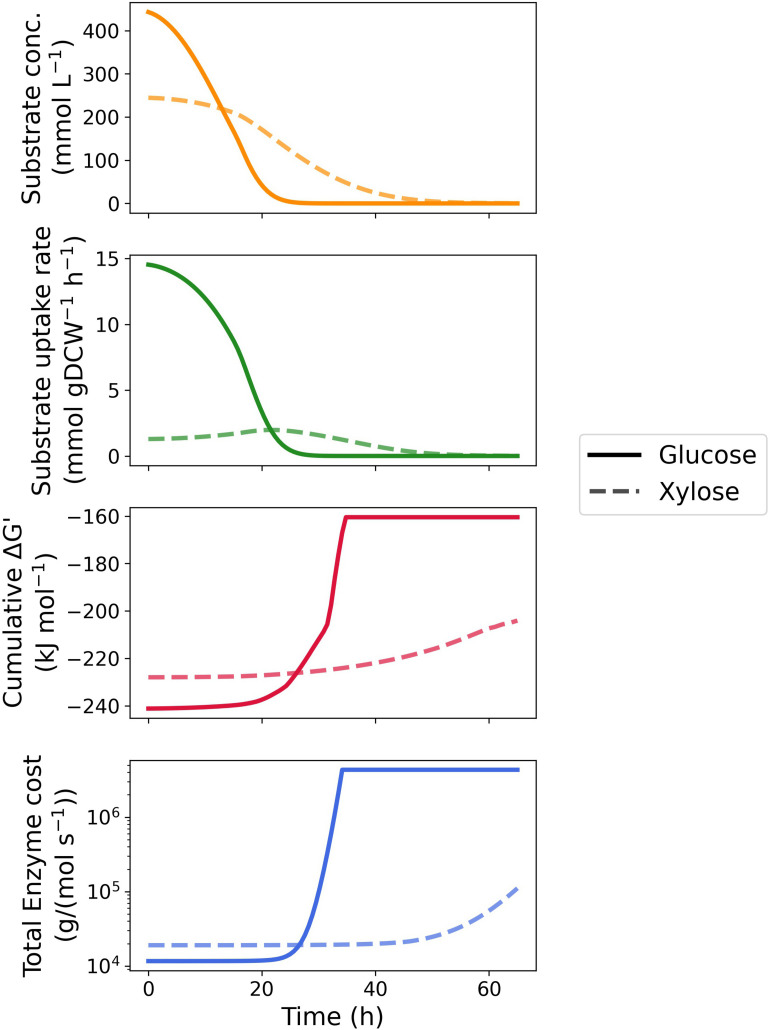
Time-course kinetic and thermodynamic profile of glucose and xylose metabolism by *Z. mobilis* BC42C Δpdc. Substrate concentrations constituted the fitting curve of experimental data. Specific substrate uptake rates were calculated as the first derivative of substrate concentrations divided by corresponding biomass concentrations. MDF optimization and enzyme protein cost estimation were performed at 100 equally spaced timepoints during fermentation with substrate concentration fixed at corresponding values. Only total cumulative ΔG′ and protein cost were illustrated, see Supplementary Figures 1, 2 for results of individual enzymes.

We then extended the MDF optimization and enzyme protein cost estimation to the entire fermentation process. As shown in Figure 3 as well as Supplementary Figures 1, 2, the MDF and protein requirements of most enzymes vary with depletion of the two substrates. With respect to glucose metabolism, the MDF of majority of the pathway enzymes rises steeply (approaching zero from the negative direction) after 25 h when the glucose concentration was below 3 mM with the exception of phosphogluconate dehydratase (edd), als and acetolactate decarboxylase (aldc) (Supplementary Figure 1A), whereas only a gentle increase was seen in enzymes located at the beginning and end of the xylose metabolism pathway (Supplementary Figure 2A). In total reaction driving force, glucose utilization maintained thermodynamic favorability over xylose until the glucose concentration was below 2 mM, when xylose achieved a maximum uptake rate.

Similar results were obtained in the time course enzyme protein requirement for both pathways. The protein cost increases drastically with the depletion of substrates to sustain a given uptake rate. As the transporter of glucose and xylose, glf is influenced the most by the substrate exhaustion since protein cost scales inversely proportional to substrate concentration. It is noteworthy that the protein requirement of als is barely affected by the substrate concentration in both pathways and dominates the total protein requirement with sufficient substrate concentrations (Supplementary Figures 1B, 2B). Like the thermodynamic driving force, glucose metabolism is always more economical in total enzyme protein than xylose metabolism until glucose is depleted.

### Growth Simulation of *Zymomonas mobilis* With Dynamic Flux Balance Modeling

To further account for cell growth dynamics and predict cellular metabolism of *Z. mobilis*, we extended the stoichiometric model from the pathway-level to the system-level and performed dynamic FBA (Mahadevan et al., 2002). In dynamic FBA, the timescales associated with cell growth and substrate uptake are assumed to be much faster than the dynamics of intracellular metabolic conversions. This a smaller dynamic system of only the extracellular metabolites to be considered while maintaining a pseudo-steady-state for intracellular metabolite concentrations for FBA. The core reaction network, consisting of 79 reactions and 70 metabolites, was built based on a genome-scale metabolic reconstructions of *Z. mobilis* (Lee et al., 2010; Pentjuss et al., 2013; Nouri et al., 2020), and includes an incomplete EMP pathway (with phosphofructokinase missing), the ED pathway, the pentose phosphate pathway, and an incomplete TCA cycle (with α-ketoglutarate dehydrogenase and malate dehydrogenase missing, Figure 1A). Estimated kinetic parameters were used to describe substrate uptake kinetics of the model. The lower and upper bounds of metabolic flux through pyruvate decarboxylase were set to zero to mimic the knockout of the associated gene.

**FIGURE 4:**
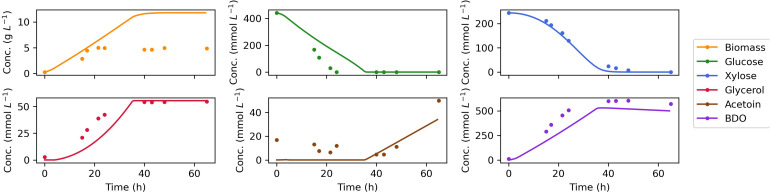
Comparison of dynamic flux balance model predictions (lines) and experimental data (dots) for *Z. mobilis* BC42C Δpdc co-utilizing glucose and xylose. The estimated kinetic parameters are used in substrate uptake kinetics. The lower bound of non-growth associated maintenance (NGAM) is set to zero. Reuptake of products acetoin and BDO are allowed for a better match of the experimental results.

The measured time course for extracellular metabolites and predictions by the dynamic FBA model for batch aerobic growth of *Z. mobilis* BC42C Δpdc on mixed glucose and xylose media are shown in Figure 4. The model simulations show good agreement with the experimental data, except for an overprediction of biomass accumulation. It could be attributable to the unmeasured metabolite leak in the fermentation. Nevertheless, the leaked metabolites will have less effect on modeling of the formation of other products since metabolic flux is additive, and the excess biomass predicted by model could have been converted into the unmeasured metabolites. Diauxic growth was not evident since the two substrates, glucose, and xylose, were consumed simultaneously. The accumulation of acetoin was delayed until glucose was exhausted, and 2,3-BDO concentration began to decrease when both sugar substrates were depleted. As a reduced product, 2,3-BDO can be re-oxidized to generate NADH, which with an external electron acceptor, can be used to produce ATP for cell maintenance. This phenomenon is implemented by addition of reuptake kinetics of 2,3-BDO in the dynamic flux balance model.

### Identification of Optimal Growth Conditions for 2,3-BDO Production

**FIGURE 5:**
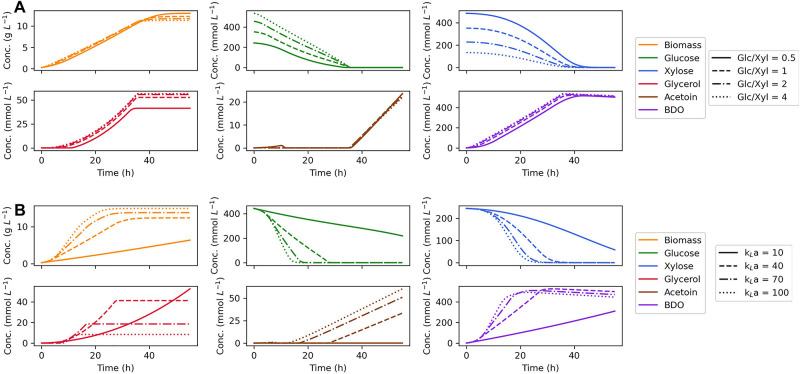
Effects of initial substrate concentration ratio **(A)** and oxygen supply **(B)** on predictions of dynamic flux balance model. In the investigation of initial substrate concentration, a gradient of glucose:xylose ratios are tested keeping the total substrate C mol as a constant. Different agitation and oxygen supply levels are studied by varying the mass transfer coefficient (k_*L*_a).

Since our dynamic flux balance model was validated to make accurate prediction of growth on dual substrates, the model was further used to identify *in silico* the optimal fermentation conditions for 2,3-BDO production. For this purpose, maximum 2,3-BDO productivity was used as the optimization objective, defined as the maximal ratio of extracellular concentration of 2,3-BDO divided by the fermentation time. First, the initial concentration of glucose and xylose was tuned to see whether a simultaneous depletion of the two sugars can be achieved. The ratio of glucose and xylose was varied from 0.5 to 4 while the total C-mol of substrates was fixed as a constant. As shown in Figure 5A, the biomass accumulates with little change between different glucose:xylose ratios; however, the consumption time of the two substrates rarely deviates either. We found that formation of reduced products, glycerol and 2,3-BDO, increase with the ratio of glucose to xylose in the media, which can be attributed to the more efficient production of reducing equivalents by glucose metabolism. Accordingly, *in silico* optimization shows that the highest maximum 2,3-BDO productivity of 14.6 mmol L^–1^ h ^–1^ at 36.2 h can be reached with a glucose:xylose ratio of 4, though the maximum productivity is insensitive to the ratio changes.

The effect of different levels of oxygen supply was also studied for which the oxygen transfer coefficient, k_*L*_a, was selected as the tuning parameter. With an increased oxygen supply, our model predicts faster growth and substrate consumption (Figure 5B). In the model, enhanced uptake of oxygen consumes extra reducing power as an effective acceptor of electrons, thereby stimulating the regeneration of redox cofactors by sugar metabolism. Changing the oxygen supply also diverts carbon flux from glycerol to acetoin and 2,3-BDO, since acetoin and 2,3-BDO are produced through the generation of NADH and NADPH, whereas glycerol production primarily serves to capture excess electrons generated through g6pdh when the cell lacks an external electron acceptor. Accordingly, the optimization of oxygen supply suggests that a high agitation rate will benefit the production of 2,3-BDO by *Z. mobilis*. On the other hand, if maximum 2,3-BDO titer is used as the optimization objective, a moderate aeration will be the optimal condition for the formation of the product which is consistent with the previous study (Yang et al., 2016b).

## Discussion

To investigate the preferable utilization of glucose over xylose by *Z. mobilis*, a thermodynamic analysis was first performed which showed a continuously stronger driving force of glucose metabolism until the substrate is exhausted. Thermodynamics is the key factor determining the feasibility and efficiency of a metabolic pathway. Rutkis et al. (2013) has reported that ATP consumption exerts a high level of control on the ED glycolysis activity of *Z. mobilis* through kinetic modeling and metabolic control analysis (Kalnenieks et al., 2014). Interestingly, our thermodynamic model also shows an enhanced driving force with a decreased ratio of ATP and ADP in both glucose and xylose metabolism pathway (Supplementary Figures 3A,B). Our results support their findings since a decreased ATP/ADP ratio usually suggests an increased ATP requirement, whereas a larger negative ΔG′ indicates a higher ratio between the forward and backward fluxes, that is, stronger metabolic activity in a pathway according to the flux-force relationship (Beard and Qian, 2007; Noor et al., 2014). Similar results were noticed by Jacobson et al. (2019) who quantified the intracellular metabolite concentrations with a ^13^C labeling approach (Bennett et al., 2008), and found a significant lower ATP/ADP ratio in *Z. mobilis* than in *E. coli* and *Saccharomyces cerevisiae*. *Z. mobilis* thus suffers an energy shortage and sustains a high ED pathway activity for ATP production (Seki et al., 1990; Kalnenieks et al., 2014; Jacobson et al., 2019). Our results also show that the ratio between reducing equivalents and their oxidized form positively correlates with the cumulative ΔG′ of both glucose (Supplementary Figures 3C,E) and xylose (Supplementary Figures 3D,F) metabolism. This suggests reducing power might have a controlling effect on *Z. mobilis* glycolytic activity as well, while more theoretical and experimental evidence is required.

Next, enzyme protein cost was estimated for both substrate utilization pathways at the initial state and during the fermentation process. A number of assumptions must be made in building the protein cost model. Besides those basic ones for modeling pathway protein cost (Flamholz et al., 2013) and those for application of the common rate law (Wu et al., 2020), two more assumptions are required for the time course analysis. First, the entire fermentation process is divided into small enough time intervals, and metabolic steady state is achieved and sustained during each interval, which means that the transition between two steady states is instantaneous. This assumption is similar to the one used in the static optimization approach to solve a dynamic flux balance model (Mahadevan et al., 2002), and thus is reasonable for our time course analysis. Second, we assume that the expression level of native enzymes is not affected by introduction of exogenous enzymes. This assumption might be violated due to interactions between the introduced enzymes and local proteins or cofactors; however, it serves as the best approximation until further experimental evidence is available. Notably, the protein cost has the unit g/(mol s^–1^), which yields mass proportion of pathway enzyme to cell dry weight multiplied by metabolic flux in mmol gDCW^–1^ s^–1^. It suggests that our time course protein cost estimation could be further validated with dynamic proteomics analyses which is, however, beyond the scope of this research.

Finally, a global stoichiometric model was constructed to simulate and optimize the growth of *Z. mobilis* by dynamic FBA. Our results show that higher 2,3-BDO productivity can be achieved with increased oxygen supply. However, fully aerobic growth for 2,3-BDO productivity is not possible since there exists a balance between excess oxygen supply where producing acetoin is favorable to 2,3-BDO and insufficient aeration that benefits the formation of glycerol. More experiment trials are therefore required to identify and specify the balance. Experimental investigation of whether increased oxygen concentrations in microaerobic fermentations improves growth rates for our engineering *Z. mobilis* strain remains as future work, as growth for ethanol-producing *Z. mobilis* typically does not benefit from oxygen availability. Taken together, with an insight of the dynamic behavior of metabolic networks, the approach shows the potential of providing profound guidance in industrial applications of biofuel production.

## Materials and Methods

### The Strain and Measurement of Growth, Substrate, and Product Kinetics in Batch Fermentations

The *Z. mobilis* strain used in batch fermentations is BC42C Δpdc which is capable of 2,3-BDO production without ethanol biosynthesis by a pyruvate decarboxylase knockout (Zhang and Himmel, 2019; Zhang et al., 2019a). The biological cultures were kept frozen in a −80°C freezer using a 20% glycerol solution. The strains were revived from frozen culture on rich media (10 g L^–1^ yeast extract, 2 g L^–1^ potassium phosphate) and 50 g L^–1^ glucose (RMG 5%) in a 50 mL baffled flask with 10 mL media. The flasks were incubated for 6 h at 30°C and 180 rpm in a shaker incubator. The revived culture was used to start the seed for the primary fermentation. The seed media was prepared in a 125 mL baffled shake flask with RMG 8% (80 g L^–1^ glucose) at a 50 mL working volume. The revived culture was transferred to the flask at a volume to achieve an initial optical density (OD) of 0.1 when measured with a spectrometer at 600 nm wavelength. The seed flask was incubated at 30°C and 180 rpm overnight in a shaking incubator. Fermentations to generate experimental design data for 2,3-BDO production were conducted in BioStat-Q plus fermentors with a 300 mL working volume. The specific fermentation parameters that were used in the described kinetic model were a constant temperature of 30°C, pH 5.8 controlled with KOH (4 N), 100 mL air flowrate through overlay rings, and an agitation at 700 rpm while glucose was present that was reduced to 300 rpm when only xylose was remaining.

Samples were taken throughout the batch fermentations for analyses. Fermentation samples were centrifuged, and supernatants were filtered through a 0.2-μm syringe filter before placing in high-pressure liquid chromatography (HPLC) vials. The samples were analyzed for carbohydrates and BDO (both Meso and SS stereoisomers), acetoin, glycerol, and other by-products. Carbohydrate analysis was done on the Shodex SP0810 carbohydrate column, and organic acids analysis was performed using the Bio-Rad Aminex HPX-87H organic acids column. Sugar utilization, 2,3-BDO, acetoin, and glycerol titers and yields were calculated based on the HPLC data.

### Estimation of Kinetic Parameters of Substrate Uptake

The glucose and xylose uptake of *Z. mobilis* is described by Michaelis–Menten kinetics with terms for their mutual competitive inhibition (Dimarco and Romano, 1985), which is formulated as:

(1) $$v_{g\,l\,c}=\frac{V_{m\,a\,x,g\,l\,c}\cdot \left[g\,l\,c\right]}{K_{m,g\,l\,c}\cdot \left(1+\frac{\left(x\,y\,l\right)}{K_{i,x\,y\,l}}\right)+\left[g\,l\,c\right]}$$

(2) $$v_{x\,y\,l}=\frac{V_{m\,a\,x,x\,y\,l}\cdot \left[x\,y\,l\right]}{K_{m,x\,y\,l}\cdot \left(1+\frac{\left(g\,l\,c\right)}{K_{i,g\,l\,c}}\right)+\left[x\,y\,l\right]}$$

where [glc] and [xyl] are extracellular concentrations of glucose and xylose, kinetic parameters V_*max,glc*_ and V_*max,xyl*_ are the maximum uptake rate of each sugar, K_*m,glc*_ and K_*m,xyl*_ are corresponding Michaelis-Menten constants that indicate the affinities of the reactants for enzyme, and K_*i,glc*_ and K_*i,xyl*_ are inhibition constants. The dynamics of substrate uptake is described with the following ordinary differential equations (ODEs):

(3) $$\frac{d\,\left[g\,l\,c\right]}{d\,t}=-v_{g\,l\,c}\cdot \left[X\right]$$

(4) $$\frac{d\,\left[x\,y\,l\right]}{d\,t}=-v_{x\,y\,l}\cdot \left[X\right]$$

where [X] is the biomass concentration, for which values were obtained by a simple linear interpolation between experimental datapoints. The ODEs were solved using odeint in the Python package scipy, and the kinetic parameters were estimated by solving the least squares problem minimizing the difference between experimentally measured and simulated substrate concentrations using openopt. The optimization was repeated several times and always converged to a stable solution. The coefficient of determination R^2^ was also calculated to evaluate the goodness of fit.

### Max-Min Driving Force Optimization and Enzyme Protein Cost Estimation

The MDF and protein cost analysis were conducted to assess the thermodynamic feasibility and protein synthesis expense of both glucose and xylose utilization pathways using a Python-based pathway analysis tool PathParser (Wu et al., 2020). The enzymes involved as well as their thermodynamic and kinetic parameters used for calculation were listed in Supplementary Tables 2, 3. Standard reaction Gibbs free energies were searched in eQuilibrator (Flamholz et al., 2012). Michaelis-Menten constants, catalyst rate constants and molecular weights of *Z. mobilis* enzymes were taken from BRENDA (Jeske et al., 2019). Geometric mean was used if multiple values were available. For heterologous proteins, parameters were taken for enzymes in their host species. Kinetic parameters for acetolactate synthase, acetolactate decarboxylase and butanediol dehydrogenase were taken from *E. cloacae* (Yang et al., 2016b), while values from *E. coli* were used for xylose isomerase, xylulokinase, transketolase and transaldolase (Zhang et al., 1995). For the substrate uptake reaction, irreversible Michaelis-Menten kinetics was applied to sugar transporter glf with zero Gibbs free energy change. During optimization, extracellular sugar concentrations were fixed at experimentally measured values, and intracellular metabolite concentrations were allowed to vary between 1 μM and 10 mM (Flamholz et al., 2013). To perform the time course MDF optimization and enzyme protein cost estimation, the batch time was divided into many time intervals in which a metabolic steady state was assumed. Accordingly, the extracellular glucose and xylose concentrations were set to the lowest values obtained by fitting in each time interval.

### Dynamic Flux Balance Model

In addition to glucose and xylose metabolism and 2,3-BDO synthesis pathways, the core metabolic reactions of *Z. mobilis* BC42C Δpdc was expanded to include an incomplete EMP pathway (with phosphofructokinase missing), the ED pathway, pentose phosphate pathway and an incomplete TCA cycle (with α-ketoglutarate dehydrogenase and malate dehydrogenase missing) (Lee et al., 2010; Pentjuss et al., 2013; Nouri et al., 2020). The constructed flux balance model consists of 79 reactions with 70 related metabolites, which is provided as Supplementary Data.

For dynamic flux balance analysis, the uptake kinetics of glucose and xylose was described by Equations 1, 2, while oxygen uptake was described by the following equation:

(5) $$v_{o_{2}}=\frac{V_{m\,a\,x,o_{2}}\cdot \left[o_{2}\right]}{K_{m,o_{2}}+\left[o_{2}\right]}$$

To better match the experimental data, reuptake of fermentation products acetoin and 2,3-BDO were allowed through the following kinetics:

(6) $$v_{a\,c\,t\,n}=\frac{V_{m\,a\,x,a\,c\,t\,n}\cdot \left[a\,c\,t\,n\right]}{K_{m,a\,c\,t\,n}+\left[a\,c\,t\,n\right]}$$

(7) $$v_{b\,d\,o}=\frac{V_{m\,a\,x,b\,d\,o}\cdot \left[b\,d\,o\right]}{K_{m,b\,d\,o}+\left[b\,d\,o\right]}$$

where [O_2_], [actn], and [bdo] are extracellular concentrations of oxygen, acetoin and 2,3-BDO, V_*max,o2*_, V_*max,actn*_ and V_*max,bdo*_ as well as K_*m,o2*_, K_*m,actn*_ and K_*m,bdo*_ are corresponding maximum uptake rate and Michaelis-Menten constants. The kinetic parameters of glucose and xylose were estimated by fitting the model to experimental data as described above. The maximum uptake rate of oxygen was assumed to be 15 mmol gDCW^–1^ h^–1^ (Mahadevan et al., 2002) and its Michaelis-Menten constant was set to 1.24 μM by experimental measurement (Balodite et al., 2014). V_*max*_ and K_*m*_ of acetoin and 2,3-BDO were assumed to be 10 mmol gDCW^–1^ h^–1^ and 5 mM, respectively.

The extracellular mass balance of above the species, as well as biomass and glycerol, are described by:

(8) $$\frac{d\,\left[o_{2}\right]}{d\,t}=-v_{o_{2}}\cdot \left[X\right]+k_{L}\,a\cdot \left[\left(o_{2}^{{}^{*}}\right)-\left(o_{2}\right)\right]$$

(9) $$\frac{d\,\left[a\,c\,t\,n\right]}{d\,t}=-v_{a\,c\,t\,n}\cdot \left[X\right]$$

(10) $$\frac{d\,\left[b\,d\,o\right]}{d\,t}=-v_{b\,d\,o}\cdot \left[X\right]$$

(11) $$\frac{d\,\left[X\right]}{d\,t}=\mu \cdot \left[X\right]$$

(12) $$\frac{d\,\left[g\,l\,y\,c\right]}{d\,t}=v_{g\,l\,y\,c}\cdot \left[X\right]$$

where k_*L*_a is the mass transfer coefficient for oxygen which is positively correlated with the impeller speed during fermentation (Mastroeni et al., 2003) and assumed to be 30 h^–1^ at 300 rpm. [O_2_^∗^] denotes the oxygen concentration in gas phase and assumed to be a constant 0.21 mM (Mahadevan et al., 2002).

### Growth Simulation

Dynamic flux balance analysis of *Z. mobilis* BC42C Δpdc was performed using the metabolic modeling package COBRApy in Python (Ebrahim et al., 2013), and was implemented in a static optimization approach. Maximization of the production of biomass and products and minimization of the consumption of substrates and oxygen were used as lexicographic constraints. Growth was simulated by integration of extracellular mass balance Equations 3, 4, 6–10 using odeint in SciPy package. All growth simulations were performed with inoculum OD of 0.84, and initial glucose and xylose concentrations of 443 and 245 mM, respectively, matching experimental conditions. The batch time for simulation was set to be 65 h, after which both substrates were depleted.

To investigate the effect of the initial sugar ratio on fermentation performance, growth was simulated at different glucose:xylose ratios of 1:2, 1:1, 2:1 and 4:1 with the total C-mol of the substrates as a constant. To investigate the effect of agitation on fermentation performance, growth simulations were performed by varying the mass transfer coefficient, k_*L*_a. The fermentation performance was quantified through the maximum 2,3-BDO productivity, defined as the maximal concentration of 2,3-BDO divided by fermentation time.

## Data Availability Statement

The raw data supporting the conclusions of this article will be made available by the authors, without undue reservation. The scripts and model used in this paper are available at https://github.com/Chaowu88/zymomonas_modeling. The pathway tool PathParser can be found at https://github.com/Chaowu88/PathParser.

## Author Contributions

CW performed the computational experiments and drafted the manuscript. RS performed the batch fermentations. ND assisted in planning the fermentation experiments. YB and PS helped plan the computational results. All authors assisted in editing the final manuscript.

## Conflict of Interest

The authors declare that the research was conducted in the absence of any commercial or financial relationships that could be construed as a potential conflict of interest.

## Publisher’s Note

All claims expressed in this article are solely those of the authors and do not necessarily represent those of their affiliated organizations, or those of the publisher, the editors and the reviewers. Any product that may be evaluated in this article, or claim that may be made by its manufacturer, is not guaranteed or endorsed by the publisher.
