# Supplementary material for: Thermodynamic and Kinetic Modeling of Co-utilization of Glucose and Xylose for 2,3-BDO Production by Zymomonas mobilis
Source: Front Bioeng Biotechnol. 2021 Jul 26;9:707749. doi: 10.3389/fbioe.2021.707749 (PMC8350737; doi:10.3389/fbioe.2021.707749)
Supplement: Supplementary file 1 [file Data_Sheet_1.docx]

**Supplementary Table 1.** Abbreviations used in the metabolic model (Fig. 1A).

| Abbreviation | Metabolite Name |
| --- | --- |
| 13DPG | 3-Phospho-D-glyceroyl phosphate |
| 2DDG6P | 2-Dehydro-3-deoxy-D-gluconate 6-phosphate |
| 2PG | D-Glycerate 2-phosphate |
| 3PG | 3-Phospho-D-glycerate |
| 6PGC | 6-Phospho-D-gluconate |
| 6PGL | 6-phospho-D-glucono-1,5-lactone |
| AC | Acetate |
| ACALD | Acetaldehyde |
| ACCOA | Acetyl-CoA |
| ACTP | Acetyl phosphate |
| AKG | 2-Oxoglutarate |
| CIT | Citrate |
| DACTYL | Diacetyl |
| DHAP | Glycerone phosphate |
| E4P | D-Erythrose 4-phosphate |
| ETOH | Ethanol |
| F6P | D-Fructose 6-phosphate |
| FOR | Formate |
| FUM | Fumarate |
| G3P | Glyceraldehyde 3-phosphate |
| G6P | D-Glucose 6-phosphate |
| GLC | D-Glucose |
| GLYC | Glycerol |
| GLYC3P | sn-Glycerol 3-phosphate |
| ICIT | Isocitrate |
| MAL | L-Malate |
| OAA | Oxaloacetate |
| PEP | Phosphoenolpyruvate |
| PYR | Pyruvate |
| R5P | Alpha-D-Ribose 5-phosphate |
| RU5P | D-Ribulose 5-phosphate |
| S7P | D-Sedoheptulose 7-phosphate |
| SUCC | Succinate |
| XU5P | D-Xylulose 5-phosphate |
| XYL | D-Xylose |
| XYLU | D-Xylulose |

**Supplementary Table 2.** Parameters used in thermodynamics and enzyme protein cost analysis of glucose utilization pathway in *Z. mobilis*. Standard Gibbs free energies (ΔG'^m^) were collected from eQuilibrator database, kinetic parameters (K_m_ and k_cat_) as well as enzyme molecular weight (MW) were chosen from Brenda. Default values of K_m_ and k_cat_ are 0.2 mM and 200 s^-1^, respectively.

| Enzyme ID | Reversibility | ΔG'^m^ (kJ mol^-1^) | Substrates | Products | Substrate K_m_ (mM) | Product K_m_ (mM) | k_cat_ (s^-1^) | Enzyme MW (kDa) |
| --- | --- | --- | --- | --- | --- | --- | --- | --- |
| glf^1^ | 0 | 0 | Glcex | Glc | 40.21 | 0.2 | 200 | 50.1 |
| glk | 0 | -12.3 | Glc;ATP | G6P;ADP | 0.2;0.63 | 0.2;0.2 | 200 | 66 |
| g6pdh | 1 | 4.4 | G6P;NADP | Gl6P;NADPH | 0.2;0.2 | 0.2;0.2 | 200 | 53.9 |
| pgl | 1 | -14.1 | Gl6P | SPG | 0.2 | 0.2 | 200 | 25.5 |
| edd | 0 | -43.1 | SPG | DDPG | 0.04 | 0.2 | 200 | 12.6 |
| eda | 1 | -1.4 | DDPG | GAP;Pyr | 0.12 | 0.2;0.2 | 200 | 66.8 |
| gapdh | 1 | 35.8 | GAP;Pi;NAD | BGP;NADH | 0.2;0.2;0.2 | 0.2;0.2 | 200 | 156.8 |
| pgk | 1 | -17.3 | BGP;ADP | G3P;ATP | 0.2;0.2 | 1.5;1.1 | 200 | 44 |
| pgm | 1 | 5.2 | G3P | G2P | 1.1 | 0.2 | 200 | 52 |
| eno | 1 | -4.1 | G2P | PEP | 0.2 | 0.2 | 200 | 45.7 |
| pyk | 0 | -33.3 | PEP;ADP | Pyr;ATP | 0.2;0.165 | 0.2;0.2 | 200 | 110 |
| als^2^ | 0 | -39.5 | 2Pyr | AcLac;CO2 | 20 | 0.2;0.2 | 200 | 190 |
| aldc^2^ | 0 | -40.3 | AcLac | Actin;CO2 | 0.2 | 0.2;0.2 | 200 | 29 |
| bdh^2^ | 1 | -23 | Actin;NADH | BDO;NAD | 0.2;0.2 | 0.2;0.2 | 200 | 100 |

^1^ K_m_ for external glucose of glf was estimated by fitting.

^2^ kinetic parameters of als, aldc and bdh were from *Enterobacter cloacae*.

**Supplementary Table 3.** Parameters used in thermodynamics and enzyme protein cost analysis of xylose utilization pathway in *Z. mobilis*. Standard Gibbs free energies (ΔG'^m^) were collected from eQuilibrator database, kinetic parameters (K_m_ and k_cat_) as well as enzyme molecular weight (MW) were chosen from Brenda. Default values of K_m_ and k_cat_ are 0.2 mM and 200 s^-1^, respectively.

| Enzyme ID | Reversibility | ΔG'^m^ (kJ mol^-1^) | Substrates | Products | Substrate K_m_ (mM) | Product K_m_ (mM) | k_cat_ (s^-1^) | Enzyme MW (kDa) |
| --- | --- | --- | --- | --- | --- | --- | --- | --- |
| glf^1^ | 0 | 0 | Xylex | Xyl | 80.96 | 0.2 | 200 | 50.1 |
| xyl^2^ | 1 | 3.7 | Xyl | Xylu | 0.2 | 0.2 | 200 | 92 |
| xk^2^ | 0 | -19 | Xylu;ATP | Xu5P;ADP | 0.28;8.2 | 0.2;0.2 | 255 | 100.5 |
| rpe | 1 | 3.4 | Xu5P | Ru5P | 0.2 | 0.2 | 200 | 23.8 |
| rpi | 1 | -1.2 | Ru5P | R5P | 0.2 | 0.2 | 200 | 17 |
| tkt1^2^ | 1 | -4.3 | R5P;Xu5P | GAP;S7P | 1.4;0.16 | 2.1;4 | 200 | 143.6 |
| tal^2^ | 1 | -0.2 | GAP;S7P | E4P;F6P | 0.27;0.285 | 0.1;1.1 | 13 | 71.6 |
| tkt2^2^ | 1 | -9 | E4P;Xu5P | F6P;GAP | 0.09;0.16 | 1.1;2.1 | 200 | 143.6 |
| pgi | 1 | -2.5 | F6P | G6P | 0.2 | 0.2 | 200 | 55.9 |
| g6pdh | 1 | 4.4 | G6P;NADP | Gl6P;NADPH | 0.2;0.2 | 0.2;0.2 | 200 | 53.9 |
| pgl | 1 | -14.1 | Gl6P | SPG | 0.2 | 0.2 | 200 | 25.5 |
| edd | 0 | -43.1 | SPG | DDPG | 0.04 | 0.2 | 200 | 12.6 |
| eda | 1 | -1.4 | DDPG | GAP;Pyr | 0.12(0.053,0.25) | 0.2;0.2 | 200 | 66.8 |
| gapdh | 1 | 35.8 | GAP;Pi;NAD | BGP;NADH | 0.2;0.2;0.2 | 0.2;0.2 | 200 | 156.8 |
| pgk | 1 | -17.3 | BGP;ADP | G3P;ATP | 0.2;0.2 | 1.5;1.1 | 200 | 44 |
| pgm | 1 | 5.2 | G3P | G2P | 1.1 | 0.2 | 200 | 52 |
| eno | 1 | -4.1 | G2P | PEP | 0.2 | 0.2 | 200 | 45.7 |
| pyk | 0 | -33.3 | PEP;ADP | Pyr;ATP | 0.2;0.165 | 0.2;0.2 | 200 | 110 |
| als^3^ | 0 | -39.5 | 2Pyr | AcLac;CO2 | 20 | 0.2;0.2 | 200 | 190 |
| aldc^3^ | 0 | -40.3 | AcLac | Actin;CO2 | 0.2 | 0.2;0.2 | 200 | 29 |
| bdh^3^ | 1 | -23 | Actin;NADH | BDO;NAD | 0.2;0.2 | 0.2;0.2 | 200 | 100 |

^1^ K_m_ for external glucose of glf was estimated by fitting.

^2^ kinetic parameters of tkt, tal were from *Escherichia coli*. Tkt involved in reactions with different reactants was marked with numbers.

^3^ kinetic parameters of als, aldc and bdh were from *Enterobacter cloacae*.


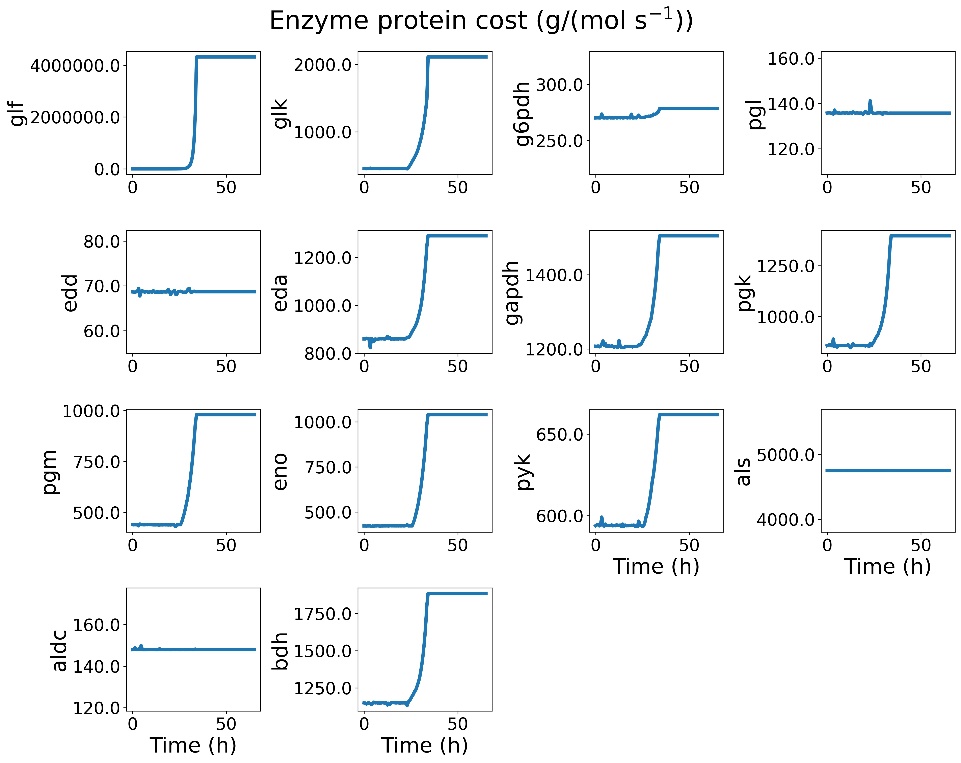
(A) (B)


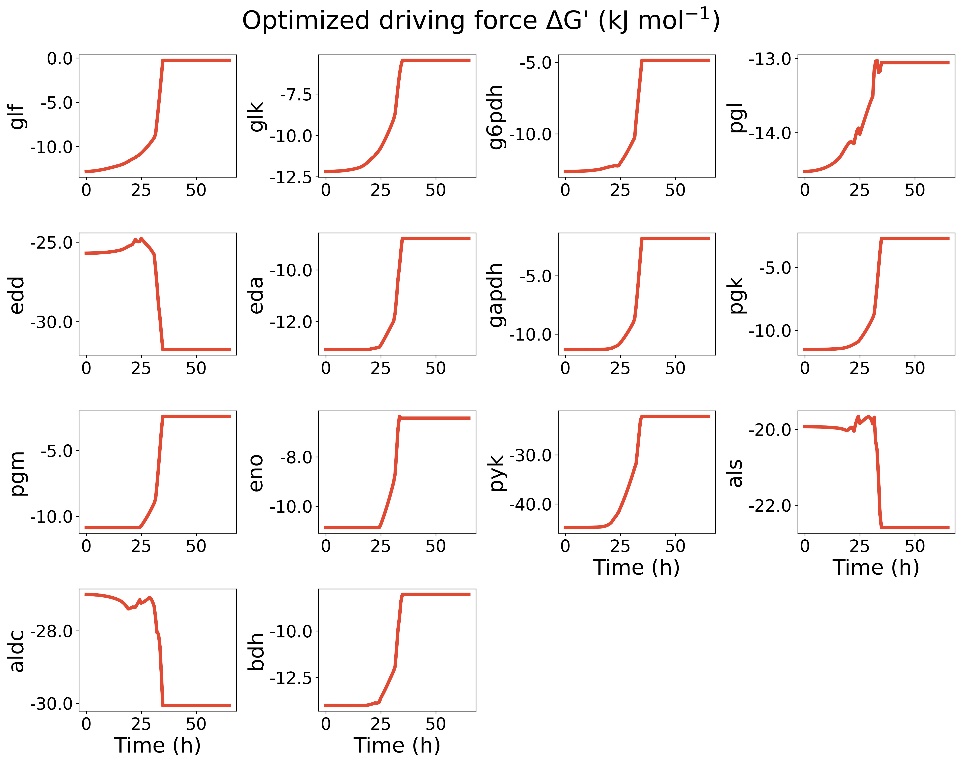


**Supplementary Figure 1.** Time course MDF (A) and protein cost (B) of individual enzymes in glucose metabolism pathway of *Z. mobilis* 42C Δpdc. MDF optimization and enzyme protein cost estimation were performed at 100 equally spaced timepoints during fermentation and extracellular glucose concentration was fixed at the estimated value of corresponding timepoint. Enzymes in the pathway were presented separately and in sum.


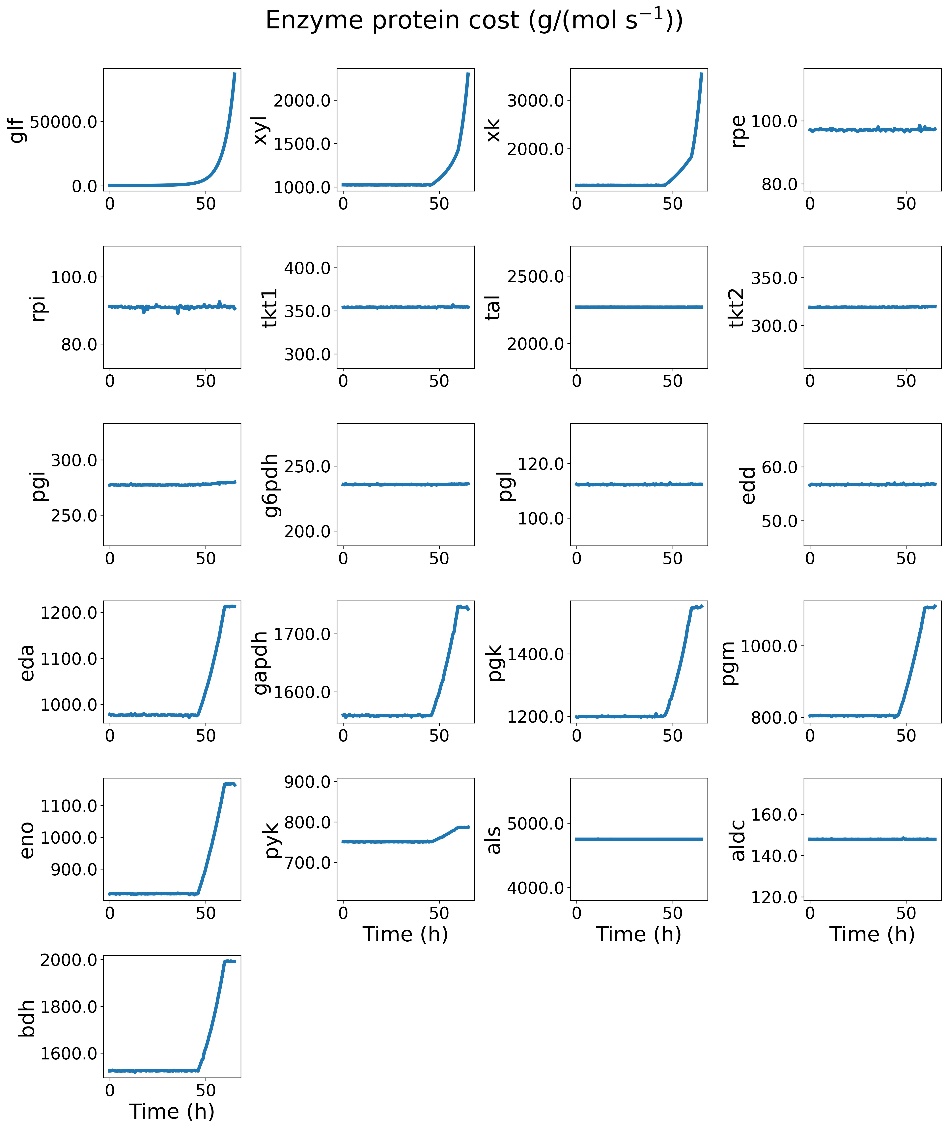

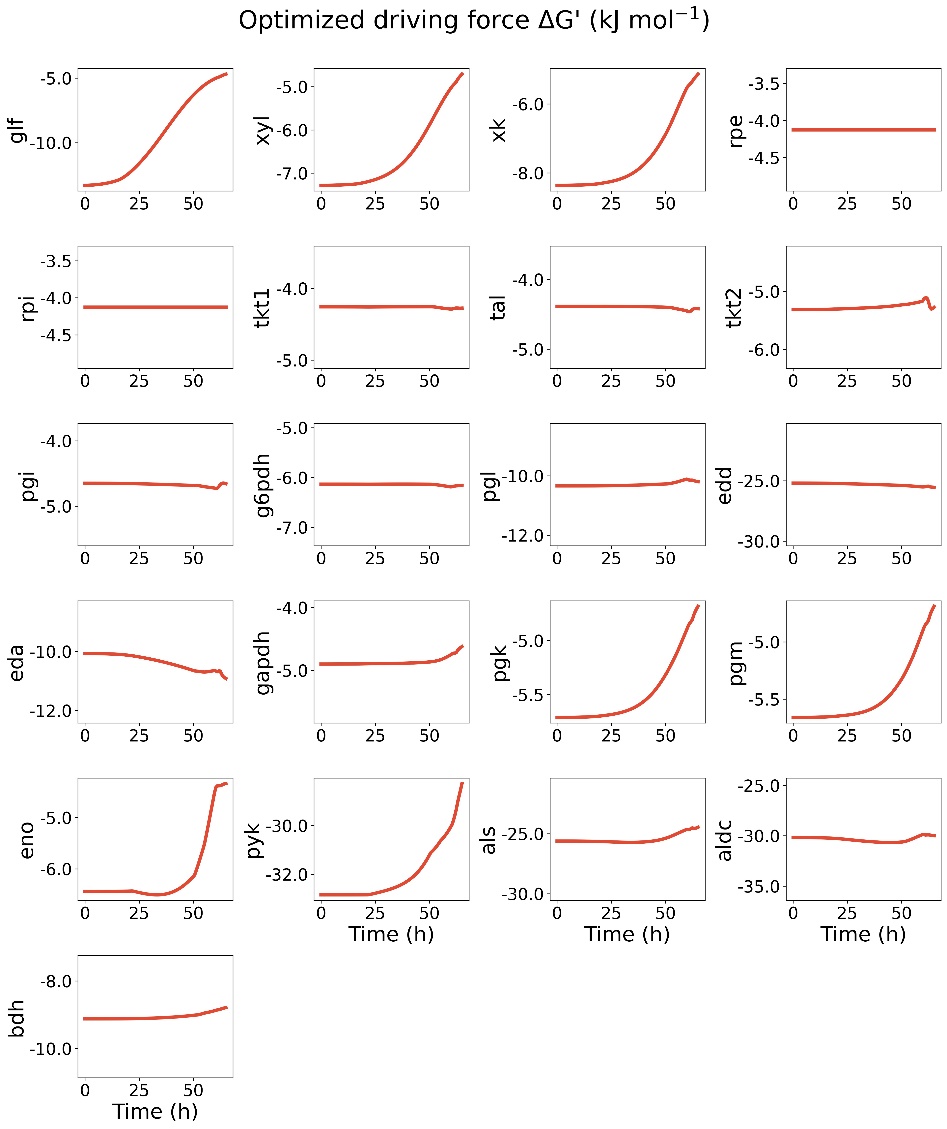
 (A) (B)

**Supplementary Figure 2.** Time course MDF (A) and protein cost (B) of individual enzymes in xylose metabolism pathway of *Z. mobilis* 42C Δpdc. MDF optimization and enzyme protein cost estimation were performed at 100 equally spaced timepoints during fermentation and extracellular glucose concentration was fixed at the estimated value of corresponding timepoint. Enzymes in the pathway were presented separately and in sum.


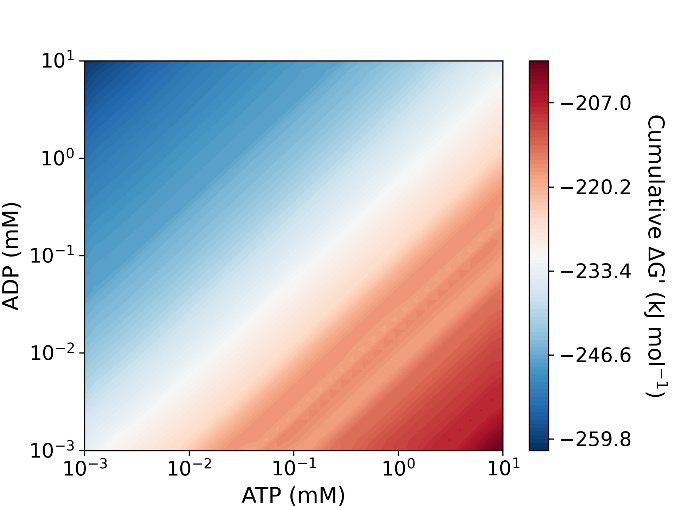

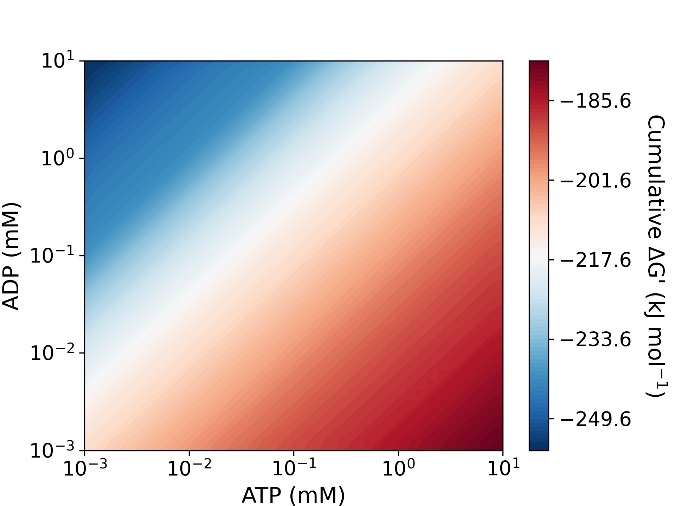
 (A) (B)


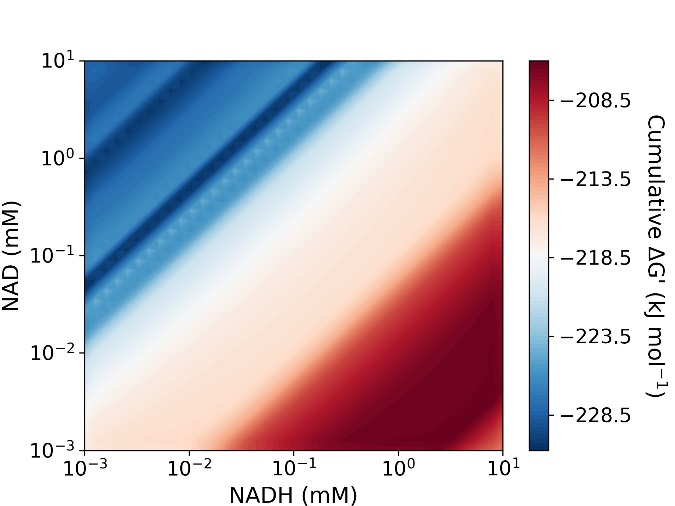

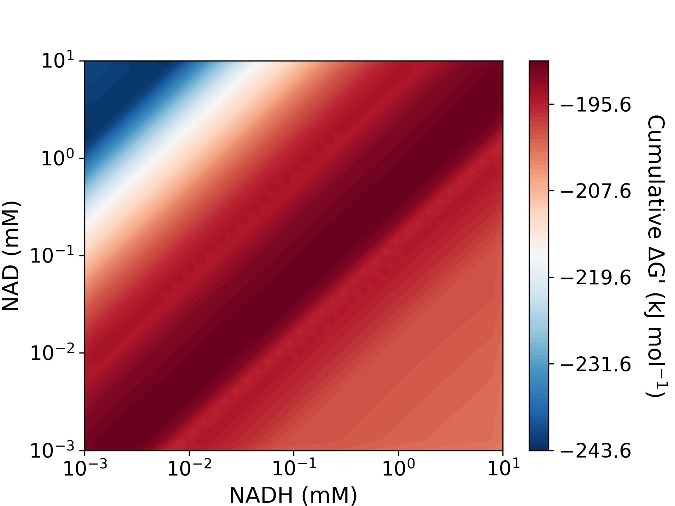
(C) (D)


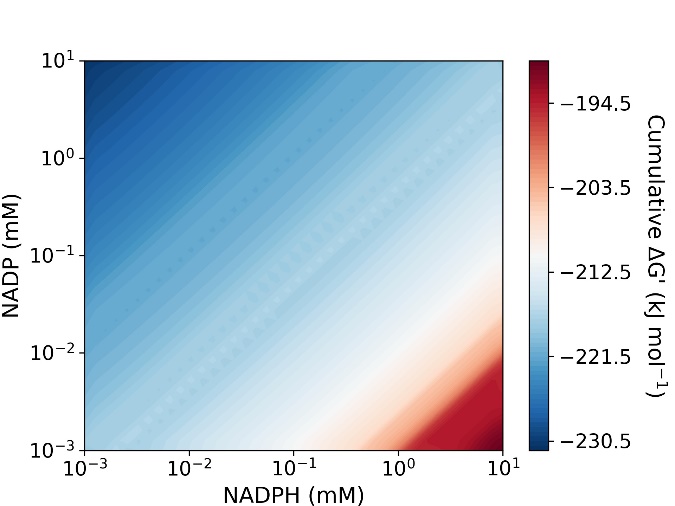

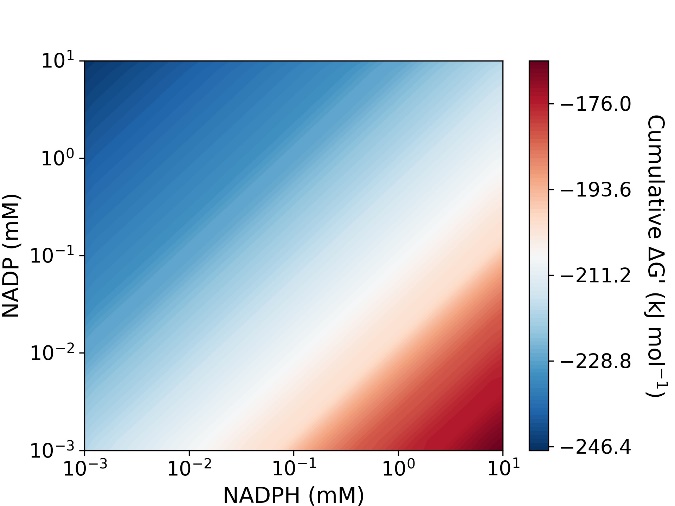
(E) (F)

**Supplementary Figure 3.** Contour of Max-min driving force (MDF) as a function of ATP and ADP, NADH and NAD as well as NADPH and NADP of glucose (A, C and E) and xylose (B, D and F) metabolism pathway. MDF optimization was performed to optimize ΔG’ of the thermodynamically least favorable reaction with metabolite concentrations constrained ranging from 1 μM to 10 mM while ATP and ADP, NADH and NAD as well as NADPH and NADP were fixed at corresponding values in the grid, respectively. Optimization results is presented as the cumulative ΔG’ of pathway reactions.
